# Helicobacter Pylori Infection in Children with Type 1 Diabetes Mellitus: A Case-Control Study

**Published:** 2016-05-24

**Authors:** Hassan Bazmamoun, Mandana Rafeey, Maryam Nikpouri, Robabeh Ghergherehchi

**Affiliations:** ^a^ Department of Pediatric, School of Medicine, Hamadan University of Medical Sciences, Hamadan, Iran; ^b^ Liver and Gastrointestinal Research Center, Tabriz University of Medical Sciences, Tabriz, Iran; ^c^ Department of Pediatric, School of Medicine, Tabriz University of Medical Sciences, Tabriz, Iran

**Keywords:** *Helicobacter pylori*, Diabetes Mellitus, Insulin-Dependent, Children

## Abstract

**Background:** *Helicobacter pylori* infection is one of the most common chronic bacterial infections. There is challenge on the real rate of prevalence of *H. pylori* in diabetic patients. This study was done to assess the prevalence of *H. pylori* infection in children suffering from type 1 insulin-dependent diabetes mellitus.

**Methods:** In this case-control study, 80 diabetic patients (as the target group) refer to the Endocrinology Clinic of Tabriz Educational and Treatment Center, Tabriz northwestern Iran and 80 non-diabetic patients (as the control group) from the group of children referring to the GI Clinic of the same center were enrolled in 2012 and 2013. Then *H. pylori* infection was assessed in two groups using measuring antibody (IgG) and stool antigen (HpSA).

**Results:** *H. pylori* infection tests were positive in 48 (60%) diabetic patients and in 32 (40%) in non-diabetic patients (*P*=0.030). There was a meaningful correlation between the frequency of *H. pylori*i and the longer the duration of diabetes (*P*<0.001). No correlation was seen between *H. pylori* infection and other factors such as age of the patients (*P*=0.840), HbA1C level (*P*=0.312), age at which diabetes was diagnosed (*P*=0.800), average daily dosage of insulin (*P*=0.232), and presence of GI symptoms (*P*=0.430).

**Conclusions:** Type 1 diabetic children especially cases with the longer duration of diabetes, are at risk acquiring *H. pylori* infection. Therefore, screening of *H. pylori* infection is helpful on the follow up of these patients.

## Introduction

Type 1 diabetes, is the most common kind of diabetes in children and one of the most common chronic diseases among children and adolescents,^2^. *Helicobacter pylori* has infected about half of the population of the world^3^ and is more common in developing countries. This infection affects people in their early age and if not treated often remains in the body for the whole life^4^.

Some studies has reported the higher prevalence of *H. pylori* in diabetic patients than the control group ^5-7^and a significant relationship between *H. pylori* infections and insulin resistance and diabetic complications^8^, but in other studies this frequency was lower^9^ and in some studies no difference with the control group was detected ^10-12^. Some authors found this association not true and to their views, other factors are responsible^13^.

The aim of this study was to assess the frequency of *H. pylori* infection in type 1 diabetic children

## Methods

In this cross-sectional study, we enrolled diabetic patients (as the target group) referring to the Endocrinology Clinic of Tabriz Educational and Treatment Center, Tabriz northwestern Iran and non-diabetic patients (as the control group) from the group of children referring to the GI Clinic of the same center in 2012 and 2013.

In the previous studies, the frequency of *H. pylori* infection among non-diabetic patients was reported as 51.2% and in diabetics 79%. With a 90% statistical power and 5% significant level, sample of 67 for each group was achieved. However, considering the possibility of loss to follow-up, the study involved 80 cases for each group^14^. Guidelines of the American Diabetes Association were used for diagnosis and clinical classification of diabetes^15^. The patients were divided and matched in 2 groups according to the age, sex, socioeconomic status.

The study was started after necessary explanation regarding the project to the parents and obtaining consent forms. All evaluations and data collection was performed by one examiner and the obtained samples were assessed by one laboratory staff. This study was performed by permission of Committee of Ethics in Tabriz Medical School of Tabriz University of Medical Sciences, Tabriz, Iran.

Five milliliters blood was obtained from basilic vein to measure *H. pylori* antibodies (IgG) (IMMUNOLAB GmbH, Frickenhausen, Germany) and sera with ODs ≥0.05 were considered positive. In addition, stool samples were collected and presence of the *H. pylori* organisms in stool was determined using a sandwich‐type EIA test (Oxoid, Cambridge, UK). According to the manufacturer's instructions, OD values of ≥0.20 were considered positive. In any case, at which both tests were positive, the patient was considered as *H. pylori* positive^16^.

Demographic findings, gastrointestinal symptoms, blood glucose and HbA1C were recorded in the information collecting form. Mean of HbA1c was calculated during one year and on the base of HbA1c, the degree of glycemic control was considered as, Good (HBA1c≤7), fair (HBA1c=7-8) and poor (HBA1c >8).

The inclusion criteria were, age less than 16 yr (for both groups) and having type 1 diabetes mellitus (for target group). The exclusion criteria from the study were history of taking antibiotic, or medications affecting the secretion of gastric acid during the previous 2 months.

Analysis of obtained data was performed by using SPSS 17 software (Chicago, IL, USA). Descriptive statistics were used to describe characteristics of the subjects, chi-square test or Fisher's exact test was used to determine the relationship between variables independently; calculation of Mean ± SE and *t*-test were used for normally distributed data. *P* value of less than 0.05, statistically, was considered significant

## Results

From 160 patients, 80 children were in diabetic group and 80 children in non-diabetic one. Thirty-two patients of diabetic group (40%) and 31(38.8%) patients of control group were male. Demographic characteristics of patients in both groups were similar (Table 1).

**Table 1:** Demographic characteristics of the diabetic patients (cases) and non-diabetic patients (controls)

**Variables**	**Cases, n=80**		**Controls, n=80**		***P*** ** value**
	**Mean**	**SD**	**Mean**	**SD**	
Age (yr)	9.48	0.52	9.25	0.36	0.940
Weight (kg)	31.66	1.92	30.8	2.09	0.670
Height (cm)	133.12	3.40	126.9	2.30	0.460

*H. pylori* infection tests in diabetic group were positive in 48 patients (60%) and in non-diabetic group were positive in 32 patients (40%) which the difference was statistically meaningful (*P*=0.030).

In diabetic group, age at diagnosis for diabetes was 7.7±0.86 yr in *H. pylori* positive group and 7.58 ±0.65 yr in *H. pylori* negative group (*P*=0.800), the average duration of diabetes was 2.72 ±0.55 yr in *H. pylori* positive group and 1.26 ±0.13 yr in *H. pylori* negative group (*P*<0.0001). The average measured HbA1C was %8±0.65 in *H. pylori* positive group and %7.9 ±0.40 in *H. pylori* negative group (*P*=0.312), and the average daily insulin dosage was 18.87±2.69‏ units in *H. pylori* positive group and 17.35 ±2.74 units in *H. pylori* negative group (*P*=0.232).‏

Therefore, in diabetic group, there was no significant correlation between positive *H. pylori* tests and variables such as age, HbA1C, age at diagnosis and daily insulin dosage, but there was a significant correlation between positive *H. pylori* infection and duration of diabetes.

In terms of relationship of GI symptoms with positive *H. pylori* tests (Table 2), there was no significant relationship in both diabetic (*P*=0.430) and non-diabetic groups (*P*=0.512).

**Table 2:** The frequency of gastrointestinal symptoms among *H. pylori* infected and non-infected patients

**Symptoms**	**Diabetic patients,** ***H. pylori*** ** infection**		**Non-diabetic patients,** ***H. pylori*** ** infection**	
	**Negative, n=32**	**Positive, n=48**	**Negative, n=48**	**Positive, n=32**
Abdominal pain	2	6	2	2
Epigastric pain	17	17	0	0
Heart burn	0	1	0	0
Diarrhea	2	3	0	2
Vomiting	7	10	2	0
Flatulence	0	3	0	0
Constipation	0	3	0	0
No symptom	4	5	44	28

## Discussion

The results of this study indicated the higher frequency of *H. pylori* infection in children affected with type 1diabetes than the control group, consistent with the results of other studies^14,17,18^. This prevalence possibly is due to increased colonization of *H. pylori* because of decreased stomach mobility and poor control of blood glucose ^5,8^. However, in some studies there has been no significant difference between *H. pylori* prevalence in both groups^19,20^and, in some, this prevalence was lower in the target group ^21,22^.

In the current study, there was no significant statistical relationship between *H. pylori* prevalence and age of the patients similar to the results of EL-Eshmawy et al study ^14^, but in some studies, prevalence of *H. pylori* was higher at older ages^23,24^. In another study, the prevalence of *H. pylori* was lower at older ages ^25^. This difference in the results is possibly due to difference in exposing people to *H. pylori* infection in different hygienic conditions.

In this study, no significant statistical relationship was seen between positive *H. pylori* tests and Hb A1C level similar to the results of Candelli et al. study^19^ but in some other studies; HbA1C range was higher in patients affected with *H. pylori* infection^14,26^.

In addition, there was no meaningful relationship between positive *H. pylori* tests and the amount of daily insulin dosage similar to Demir et al. study ^27^. Therefore, according to the findings of this study, *H. pylori* infection has had no effect on control of blood glucose in these patients. However, insulin need in diabetic patients infected with *H. pylori* was higher ^14,28^. Insulin need in diabetic patients infected with *H. pylori* was lower^29^. To better judgment, other influential factors on blood glucose such as diet and patients’ compliance should be considered as well.

In the current study, there was a meaningful association between positive *H. pylori* tests and duration of diabetes consistent with the results of other studies ^14,25,30^. However, in Mehmet Demir et al study, there was no association between these two^27^.

In terms of association between GI symptoms and positive *H. pylori* tests, no meaningful association was seen in this study consistent with the results of previous studies ^14,20, 31^. The prevalence of GI symptoms in diabetic patients affected with *H. pylori* infection has been higher ^30, 32^.

Therefore, according to the results of this study, in diabetic patients even if there are no clinical symptoms related to *H. pylori* infection, this infection should be considered particularly if the duration of diabetes is longer.

The present study had two limitations, the number of cases was small and the study period was short.

## Conclusions

Type 1 diabetic patients especially who have long-lasting disease are at more risk to acquire *H. pylori* infection. Regarding of additional problems, which this infection can create for these patients, considering this issue and screening for *H. pylori* infection can be helpful in follow up of children suffering from type 1 diabetes.

## Acknowledgments

The authors are grateful to all the participants in this study for their cooperation.

## Conflict of interest statement

Researchers declare no conflict of interest.

## Highlights

- Prevalence of H. Pylori infection is higher in children with type 1 diabetes mellitus.
- There is no correlation between H. Pylori infection and glycemic control in diabetic patients.
- There is no association between H. Pylori infection and presence of gastrointestinal symptoms in diabetic children.
- Type 1 diabetic children with longer duration of the disease are at higher risk to H. Pylori infection
